# Telepathology in Nigeria for Global Health Collaboration

**DOI:** 10.5334/aogh.3673

**Published:** 2022-09-16

**Authors:** Olugbenga Akindele Silas, Fatimah Abdulkareem, Jorge Eduardo Novo, Yinan Zheng, Drew R. Nannini, Demirkan B. Gursel, Rose Anorlu, Jonah Musa, Firas H. Wehbe, Atiene S. Sagay, Folasade T. Ogunsola, Robert L. Murphy, Lifang Hou, Jian-Jun Wei

**Affiliations:** 1 Department of Pathology, College of Health Sciences, University of Jos, Nigeria; 2 Department of Anatomic and Molecular Pathology, College of Medicine, University of Lagos, Lagos, Nigeria; 3 Department of Pathology, Feinberg School of Medicine, Northwestern University, Chicago, USA; 4 Center for Global Oncology, Institute for Global Health, Feinberg School of Medicine, Northwestern University, Chicago, USA; 5 Center for Population Epigenetics, Robert H. Lurie Comprehensive Cancer Center and Department of Preventive Medicine, Northwestern University Feinberg School of Medicine, Chicago, USA; 6 Department of Obstetrics and Gynecology, College of Medicine, University of Lagos, Lagos, Nigeria; 7 Department of Obstetrics and Gynecology, College of Health Sciences, University of Jos, Nigeria; 8 Department of Surgery, Division of Cardiac Surgery, Feinberg School of Medicine, Northwestern University, Chicago, IL; 9 Department of Microbiology and Parasitology, College of Medicine, University of Lagos, Lagos, Nigeria

**Keywords:** telepathology, Nigeria, pathology services, resource-limited settings

## Abstract

Inadequate pathology personnel and high cost of running a Pathology facility are factors affecting access to timely and quality pathology services in resource-constrained settings. Telepathology is a novel technology that allows Pathologists to remotely assess collected samples. Though the initial cost of setting up a telepathology facility is high, its overall benefits far outweigh the cost. Its usefulness as a quality assurance measure, as a permanent image data storage system, in reducing costs associated with repeated slide preparations, reducing turn-around time of pathology reports, in collaborative research and in teaching has been well documented. This paper highlights the experiences, gains and challenges encountered in the deployment of telepathology in two resource-constrained settings in Nigeria. Overcoming the challenges associated with setting up a telepathology service in sub-Saharan Africa is important as it has the potential to improve overall health outcomes in a medically underserved region while ensuring technology and knowledge transfer are achieved.

## Introduction

Telepathology services in West Africa are grossly inadequate despite scare pathology facilities and pathologists in this region [1]. It is documented that in Africa, pathology-core services are hampered by lack of equipment, inefficient processes and inadequate personnel [1,2]. An earlier study reported the pathologists to population ratio is in excess of 1 to 2.5 million people in most regions in Africa [1,2]. In Nigeria there are approximately 105 pathologists for an estimated population size of 200 million people, supporting the pathologist to population ratio reported previously [3,4]. However, this ratio is insufficient to meet the needs of quality and timely pathology services [1,3]. Consequently, a small percentage of cancers are confirmed by pathologists, resulting in clinicians making medical decisions without pathology reports, which often leads to poor treatment outcomes as a result of misdiagnosis [3,4,5].

Several attempts have been made to institutionalize telepathology in Sub-Saharan Africa, focusing primarily on teaching and research purposes [3,4]. In 2013 at the Pathology Laboratory in Kamuzu Central Hospital (KCH) in Lilongwe Malawi telepathology service was institutionalized to support local pathologists, for both research and clinical care [5,6]. This has led to improved pathology service for the region [3,4,5,6,7,8]. Functional telepathology service is however currently unavailable in West-African countries including Nigeria, the most populous country in Africa [1].

Telepathology involves the use of telecommunications to send image-rich data between remote locations for clinical diagnosis, education, and research [6]. Generally, there are four basic platforms for telepathology: (1) static images, (2) whole-slide scanning, (3) dynamic non-robotic tele-microscopy, and (4) dynamic robotic tele-microscopy [8,9]. The static images requires appropriate selection of relevant diagnostic fields and the use of limited technical infrastructure (internet connection, a microscope and a digital camera) which are of great benefit. This method, however, requires technical knowledge on appropriate fields for capture. It differs from whole-slide imaging systems which allows the pathologist to see the entire specimen at a range of magnifications. This obvious advantage comes with considerable costs which includes the purchase of slide scanning equipment, increased information technology (IT) support, and server space to allow data storage. A system that provides the benefit of whole-slide review with simpler technology at a much lower cost than whole-slide imaging systems is the dynamic non-robotic tele-microscopy. It involves the transmission of video images across any of several internet-based teleconference systems, however, it requires a skilled local pathologist to maneuver the microscope, depends on image resolution, camera quality and internet speed. Finally, a system that allows the consulting pathologist to control the objective and stage of microscopes remotely is known as the robotic tele-microscopy, which is often technically challenging and costly [7,8,9,10,11].

Currently, the cost of pathology service in Nigerian Government facilities ranges from $10 US to $20 US (unpublished estimate) due to repeat sections and occasional Immunohistochemistry (IHC) requests, a service that is beyond the reach of the majority of the population. These facilities are available mainly in tertiary health centers and a few private laboratories located in the large cities distant from the general population in villages and local communities. Delay in obtaining reports with occasional misdiagnosis or incomplete diagnosis by local pathologist has resulted in poor treatment outcomes. Therefore, there is a dire need for a sustainable system or model that can provide second opinion through remote consultations. The purpose of telepathology was to generate high quality virtual images, improve turn-around time for accessing images remotely and promote team review of target disease, correct interpretation of pathology and arriving at consensus for clinical and research use. This will be achieved through robust international consultations and clinical support in addition to achieving training and research to improve local health outcomes.

## Methods

### Establishment of basic telepathology at two Nigerian sites

Leveraging on existing international research collaboration between North-western University (NU) and two Nigerian institutions: Jos University Teaching Hospital (JUTH) and University of Lagos Teaching Hospital (LUTH). We obtained an internal grant from the Northwestern Harvey Institute for Global Health for the purchase of telepathology facilities for the two participating institutions. Novel to this study was the use of portable digital slide scanner (Grundium Ocus MGU-00003), a soft-ware image viewer (Aperio Leica RUO version 12.4.3.5008) and a cloud server with secured password for uploading, transferring and storing of images. To achieve a seamless process doctors, technicians and computer operators from the two Nigerian institutions attended training sessions on installation and process of the system, and how to resolve challenges. Institutional support was obtained from the leaderships of the institutions to develop telepathology operational policies for patient care so as to ensure its sustainability locally.

### Establishment of training platform and standard procedure for Nigeria-Northwestern telepathology network

Before the hands-on telepathology procedure, the Northwestern University (NU) Laurie Cancer Center pathologists first trained pathologists, residents, and lab technicians at two large Nigerian collaborating hospitals, Jos (JUTH) and Largos (LUTH) University Teaching Hospitals using a total of five virtual training sessions with question-and-answer components. The standard procedure that was established include proper handling of instruments, imaging scanning and review, patient’s deidentification procedure, secured data storage, data sharing, imaging reading and interpretation. Our immediate goal was as a collaborating pathology team (LUTH, JUTH and NU) to evaluate all collected research cases from the Nigerian pathology facilities to meet our current NIH funded research grant objectives as well as improve the diagnostic accuracy at the Nigerian hospitals.

### Application of a digital whole-slide scan system

With a digital slide scanner, digital images of a whole slide at multiple magnifications were made. These files were stored on a computer or external storage device before uploading to a secure site thus creating a local copy that can be viewed offline after file synchronization between the three collaborating sites. The workings of this system were used in achieving the path-core goals for the on-going National Institutes for Health (NIH) HIV and malignancies grant (NIH U54CA221205 and R21TW12092 grants) where cervical cancer lesions were processed at the two collaborating Nigerian institutions (JUTH and LUTH), scanned, stored and uploaded for viewing by the international collaborators at NU.

## Results and Discussion

### Telepathology at JUTH and LUTH

**Figure 1:**
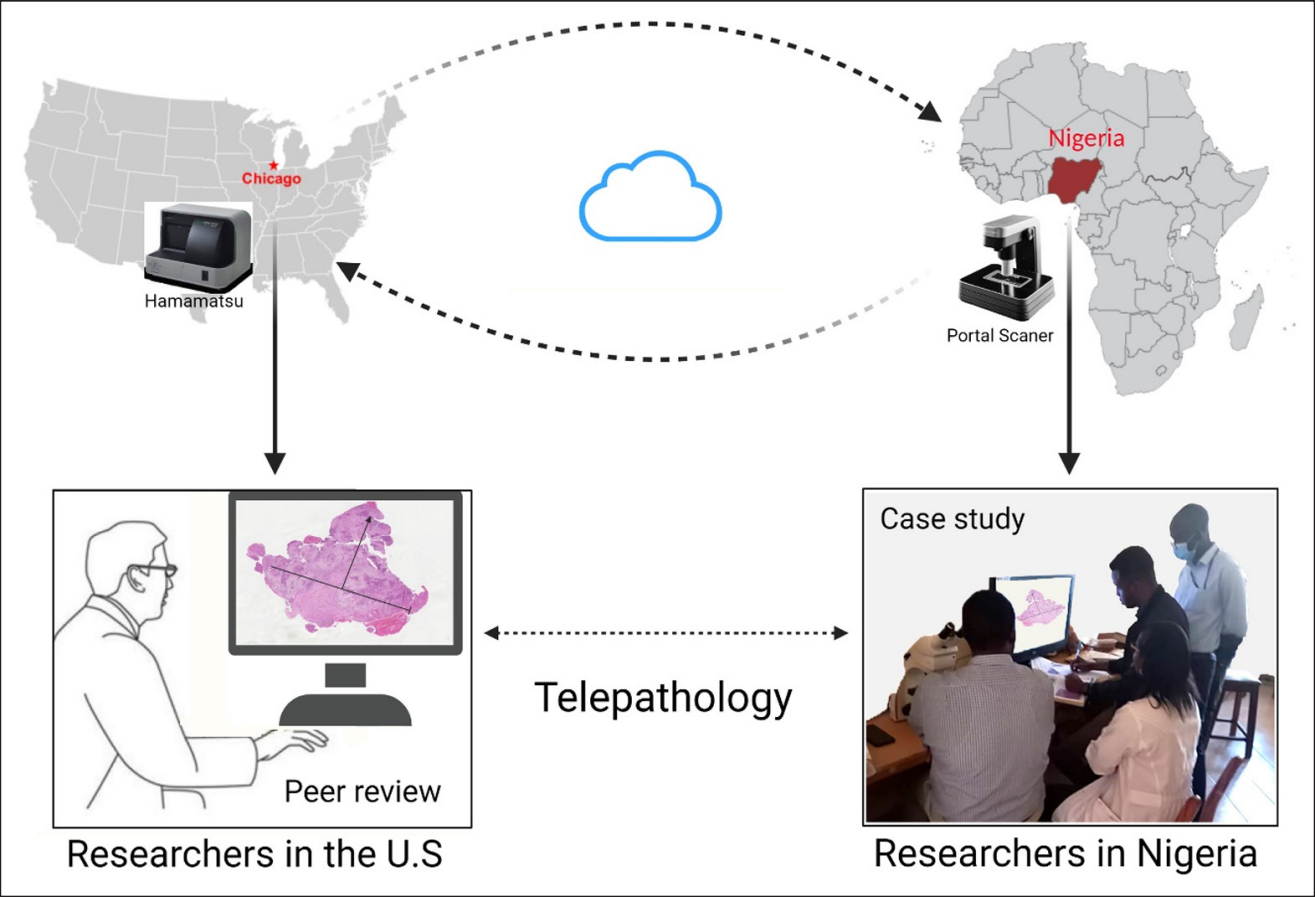
Slide images that have been scanned and uploaded in a cloud sever are viewed remotely by local pathologists in Nigeria and their international collaborators in the US.

**Figure 2:**
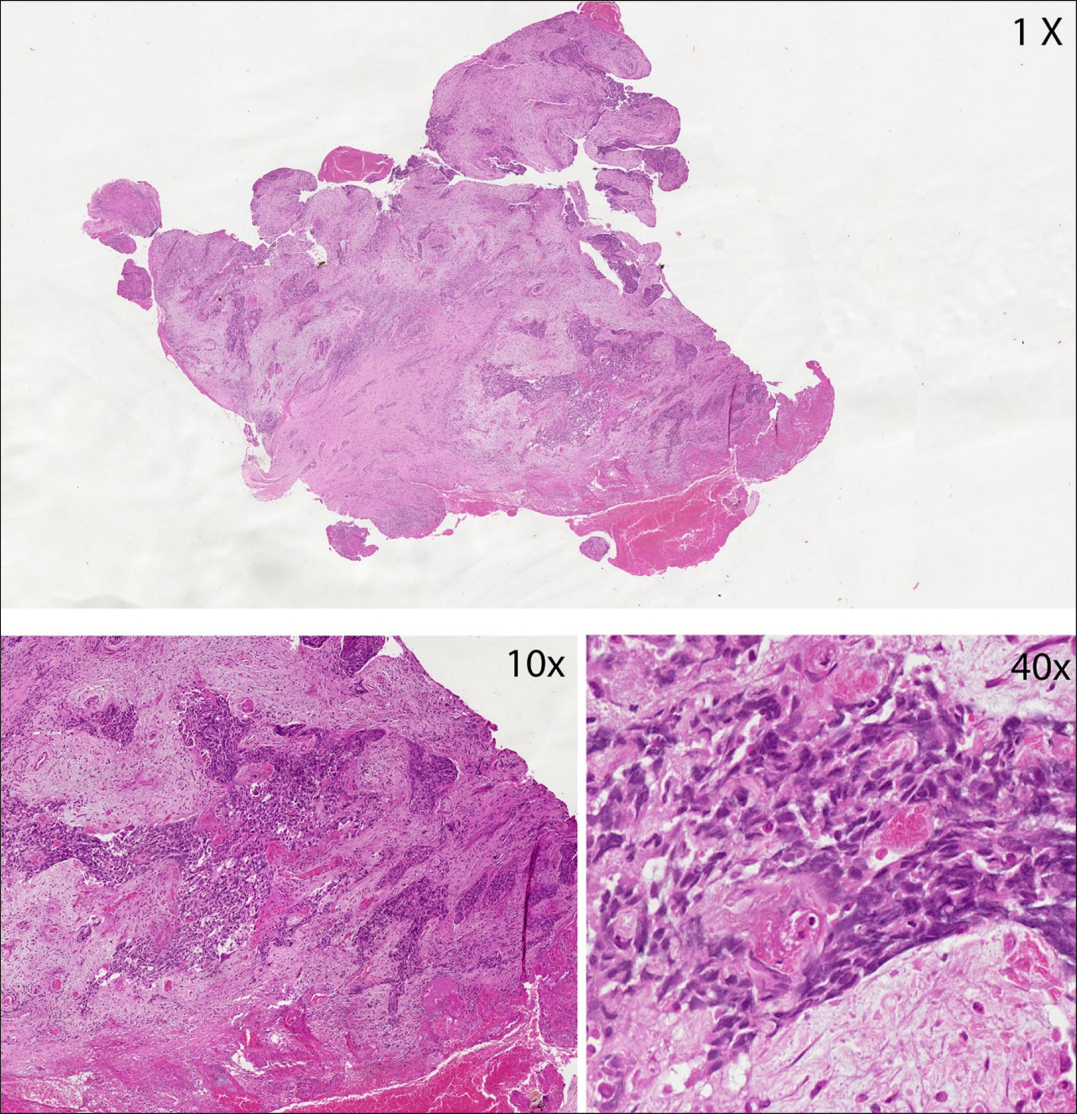
Scanned virtual image of invasive cervical cancer and viewed at low and high resolution.

Digital imaging services are now becoming an important integral component of our workflow. It has been found to be useful for patient enrollment, digital archiving, and pathology review purposes in the U.S. and other western countries [9,10,11,12,13,14,15]. Digital central pathology review and quality assurance activities of our Nigerian slides are now routinely performed at Northwestern University (Figure 1). Digital pathology has been used extensively for pathology review of cervical cancer cases for our U54 research project enrollment (Figure 2).

We have also begun the process of scanning sets of Hematoxyline and Eosin (H&E) slides guided by a set of protocols, so as to have high-quality image sets that will be linked to REDCap for future image analysis (IA) and validation projects which are currently being planned. The use of this technology provides many advantages including: superior quality to static images, eliminates distribution of glass slides, solves logistical issues with rapid turnaround times, reduces costs of tracking and loss or broken slides, and is accessible anytime via the Internet and offline after download.

A digital pathology review application facilitates a complete digital pathology review process and provides a digital mechanism for expert pathologists to review cases consisting of digital images, pathology reports, and custom-built case review forms. Our established imaging infrastructure has the capacity to generate up to 50 whole slide images at 40x magnification per day. All electronic histological image data generated by local sites were stored and served from a cloud-based server. The telepathology system is an invaluable medical resource for us. With the inadequate ratio of pathologist per population in most African countries worsened by lack of pathologist’s specialization, telepathology has become a tool that will revolutionize pathology services thus improving clinical care in most African countries [13,14,15,16].

In the past two years, over 200 cases of cervical cancer slides collected from JUTH and LUTH were scanned and analyzed through this process. Digital slides were reviewed by pathologists from the three collaborating institutes (JUTH, LUTH and NU). The advantages of this system were that we were able to: 1) arrive at consensus of pathohistology analysis and evaluation, including tumor type, grade and differentiation; 2) accurately measure the tumor size, dimension, extension, necrosis, and other pathologic features; 3) capture high quality screen shot images for publication, education and illustration; 4) reach a fast turnaround time of 3 to 5 days as opposed to a few months for inter institute slide evaluation; and 5) scanned virtual slides have become valuable educational materials for local pathologist, researcher and trainees who can easily access these deidentified virtual slides.

It is hoped that this current telepathology program developed and deployed in Nigeria can provide a model for other West-African countries, as well as other countries worldwide. The initial high cost for purchasing and installing of the equipment and that of training cannot be compared to its eventual positive impact on health outcomes and the knowledge transfer gained. The usual unquantifiable large cost for specialist training abroad, for slide and tissue transportation and the expense associated with repeat cut sections, the cost of delayed/inaccurate pathology diagnosis and the attendant cost of adverse health outcomes are now reduced.

### Impact of telepathology on diagnosis, medical research, and global health training and education

The internet telecommunication personnel were on ground to train and guide us through the installation and deployment of the scanner and viewer. Subsequently, we obtained second opinions from international colleagues thus improving the quality of clinical diagnosis. We have noticed a reduction in turn-around time of pathology reports which has improved quality assurance due to multiple consultations. This is vital for the diagnostically difficult cases. It has afforded our local staffs (pathologist, resident doctors, and laboratory technician) the opportunity for well needed improvement in their knowledge and skills from our international collaborators. Our government and institutions are gaining high-quality knowledge without the expense of overseas travel and lodging.

With improved qualitative and timely diagnosis, clinicians now make patient’s management decisions based on timely and reliable pathology reports. This will invariably improve clinical outcomes of our patients. With our current international collaboration and improved quality of our reports, we expect better health outcomes, and our local institutions will improve their reputation by producing quality pathology reports comparable to that obtained in developed countries. This will improve patronage and clientele.

International collaborative research is now easier, less expensive and seamless. The transportation of tissues for further analysis is no longer needed as digital slides of comparable quality and representation can be viewed remotely in a synchronous or asynchronous manner. Hopefully with the proliferation of such telepathology services in many countries in Africa and continued collaboration from developed nations, an overall improvement in global health will be achieved through reduced disparities in efficient and quality pathology services.

### Challenges

Although the installation of the scanner and viewing software was uncomplicated, several challenges can be faced in building telepathology system in developing countries. Most image view software can be access freely online, selection of the right software to match with different scanners can be problematic. This requires consulting with vendors and experienced pathologists and to find one that fits your needs. For example, we initially considered NDP2 viewing system used by the Northwestern team. It costs approximately $1,000 US for access lasting one month which required monthly renewal for the two sites. With the NDP2 software also came the challenge of image formats (SVS, NDP and TIF). The NDP2 software could only view images saved as TIF format, but these images were not stable, and the sharpness lost over time. We later resorted to the use of Aperio (Leica RUO version 12.4.3.5008) viewer which came with a free online download.

The key instrument for telepathology is a scanner which costs $14,500 US dollars each for the two Nigerian sites. Other costs include external storage and web cloud server.

Another challenge faced was the time for scanning and uploading of images into Dropbox or other commercial iCloud. It took 5 to 10 minutes to scan a slide depending on the tissue size but might take 10 to 30 minutes to upload each image to Dropbox depending on image size and internet quality. For a seamless process, we obtained a router with internet data worth approximately $15 US dollars for a session lasting 15 to 20 hours. This may add some unbearable costs for undeveloped regions and hospitals.

A constant power source was mandatory for a seamless process during scanning and uploading onto the cloud server. This is a major challenge in our setting as we had to augment the local power source with external diesel-powered generators.

Transition from a conventional pathology platform to telepathology platform was time consuming at the beginning and it requires the training for all key personnel involved in the process for efficiency and effectiveness including laboratory technicians, resident doctors, pathologists and IT personnel. Continuous education of all personnel involved helped address these issues.

Lastly, most countries are yet to give clear-cut regulations regarding the liability of physicians delivering care across different institutes or internationally recognized borders. However, encrypting patients’ data on the Internet will limit the possibility of legal issues. Diagnostic support rather than consultation is currently being upheld.

In the future, the possibility of integrating smart phones, mobile devices and other multimedia to this system can make telepathology an easy, economically viable tool for promoting pathology practice, medical education and research. There remains a need to be vigilant for government regulations regarding the protection of the human subject’s information through trans-border digital pathology. Furthermore, research into cost-to-benefit analysis, effects on the development of local expertise and improvement in service utilization over time to access progress achieved should be carried out and published.

## Conclusion

We report and provide our experience, challenges and gains with the installation and use of telepathology equipment for research, training and clinical care in Nigeria by networking with Laurie Cancer Center pathologists at Northwestern University in the United States. Though associated with an initial high cost, the equipment and processes involved is cost-saving in the long run and revolutionary in positively impacting health outcomes and enhancing research in resource-constrained settings where scare pathology services predominate. Policy makers in resource-constrained settings should consider telepathology as necessary in-patient care, thus investing in this critical technology to improve its health indices.
